# Heat Treatment and Formation of Magnetocaloric 1:13 Phase in LaFe_11.4_Si_1.2_Co_0.4_ Processed by Laser Beam Melting

**DOI:** 10.3390/ma13030773

**Published:** 2020-02-07

**Authors:** Jwalant Kagathara, Sandra Wieland, Eric Gärtner, Volker Uhlenwinkel, Matthias Steinbacher

**Affiliations:** 1 Leibniz Institute for Materials Engineering—IWT, Badgasteiner Straße 3, 28359 Bremen, Germany; e.gaertner@iwt-bremen.de (E.G.); uhl@iwt.uni-bremen.de (V.U.); steinbacher@iwt-bremen.de (M.S.); 2 Fraunhofer Institute for Manufacturing Technology and Advanced Materials IFAM, Wiener Straße 12, 28359 Bremen, Germany; sandra.wieland@ifam.fraunhofer.de; 3 Faculty of Production Engineering, University of Bremen, Badgasteiner Straße 1, 28359 Bremen, Germany

**Keywords:** magnetocaloric materials, LaFeSiCoalloys, laser beam melting, heat treatment, magnetocaloric properties

## Abstract

In recent years, magnetocaloric materials have been extensively studied as materials for use in alternative cooling systems. Shaping the magnetocaloric material to thin-walled heat exchanger structures is an important step to achieve efficient magnetocaloric cooling systems. In the present work, experimental investigations were carried out on the heat treatment of LaFe_11.4_Si_1.2_Co_0.4_ alloy processed by Laser Beam Melting (LBM) technology. Due to the rapid solidification after melting, LBM results in a refined micro structure, which requires much shorter heat treatment to achieve a high percentage of magnetocaloric 1:13 phase compared to conventional cast material. The influence of the heat treatment parameters (temperature, time, and cooling rate) on the resulting microstructure has been extensively studied. In addition to the conventional heat treatment process, induction technology was investigated and the results were very promising in terms of achieving good magnetocaloric properties after short-time annealing. After only 15 min holding time at 1373 K, the magnetic entropy change (∆S) of -7.9 J/kg/K (0–2 T) was achieved.

## 1. Introduction

Magnetocaloric materials and systems have attracted high interest in research due to their potential to provide a more efficient and environmental friendly alternative to conventional compressor-based cooling devices [1]. In past years, many magnetocaloric materials have been discovered, which exhibit magnetic field dependence of the structural transition, which generally results in a giant magnetocaloric effect (MCE). The Gd5Si2Ge2-based compound shows an entropy change (∆S_M_) of around 18 J/kg/K, which corresponds to almost double compared to the ∆S_M_ of gadolinium, when changing the magnetic field from 0 to 5 T [2]. However, the possible applications for this material are very limited due to the high cost and low availability of gadolinium and germanium. In recent years, the compounds of LaFeSi and MnFePX (X = Ge, Si) as well as Ni-Mn-based Heusler alloys have proven to be particularly promising for a broader application. Especially La(FeSi)_13_-based alloys came into focus during the last years, since they show high magnetocaloric effect in a broad temperature range and are at the same time composed of relatively cheap and abundant elements [3]. The transition temperature or Curie temperature T_C_ at which the maximum MCE occurs can be adjusted over a wide temperature range by alloying with Co, Ce, or Mn or by introducing interstitial hydrogen. However, the difficult phase adjustment and the associated complex material production processes and the brittleness of the material complicates the shaping and thus makes it problematic for use in magnetocaloric cooling devices.

The first samples of La(FeSi)_13_-based alloys were produced by melt metallurgy. However, when cooling from the melt, α-iron is formed in addition to the desired 1:13 phase. So, to achieve the desired magnetocaloric 1:13 phase, tempering for several days at temperatures above 1100 ° C is necessary which makes the process uneconomical [4,5,6]. As an alternative, powder metallurgy manufacturing methods have been used in which the alloying elements are available as powders, which are then mixed, milled, pressed, and then reactively sintered for usually four to eight hours [7,8]. Using this process, the temperature range in which α-iron forms is avoided and the desired phase is directly achieved. However, this process is also not suitable for industrial production due to the high effort and many process steps. Alternatively, it was possible to show on the basis of melt spinning that the required soak time can be significantly reduced by rapid quenching from the melt [9]. The diffusion paths for setting the phases during the heat treatment are thus shorter. However, since the strips produced by melt spinning still have to be milled before the subsequent shaping processes, gas atomization is much more suitable as a rapid quenching process for large-scale material production. This process directly produces spherical metal powder, the size distribution of which can be tailored to the subsequent shaping process using the respective process parameters.

To actually produce efficient magnetocaloric cooling devices, the materials must be formed into thin platelets or complex block structures with regularly distributed microchannel [10]. Various methods have already been tested to achieve these complex structures with La (Fe, Si) 13-based alloys. The authors in [11] used hot pressing technique to compress the powder material. The porosity resulting from this process has a positive effect on the mechanical properties and reduces the hysteresis. However, the freedom of form is severely restricted in this process, and the desired fine structures or thin channels cannot be produced. Another concept is the processing of a polymer-bound material. In this process, LaFeSi powder is mixed with approximately 3–10 wt. % polymer and can be shaped, for example, by extrusion. However, the entropy change based on the volume and also the thermal conductivity are reduced in accordance with the volume fraction of the polymer [12,13,14]. Extrusion with subsequent sintering process has been also tested on LaCe(FeMnSi) alloys [15]. Comb-like structures with a wall thickness of 300 µm could be produced, which are very suitable for use in cooling devices.

Additive manufacturing (AM) of magnetocaloric materials has gained immense momentum in recent decades. The advantage of this technology, besides the high geometrical freedom, is the rapid cooling of the material after melting. It is well established that rapid solidification processes lead to a refined microstructure, which is potentially reducing the heat treatment time to achieve the 1:13 phase to several hours [3,12]. Zhang et al. [16] produced different samples of Fe-30 at. % Ni using selective laser melting technique by varying laser scanning speed. It was found that saturation magnetization increased from 0.1 to 0.4 m/s but decreased again at scanning speeds greater than 0.4 m/s. In [17,18], the authors have demonstrated the use of the Laser Engineered Net Shaping (LENS) technique for fabricating components of different magnetic alloys and have shown promising magnetic properties of the samples. Laser Beam Melting (LBM) technology was first applied to La(Fe,Si)_13_-based alloys in [19]. The main disadvantages reported by Moore et al. [19] is achieving the minimum channel diameter of 800 µm and formation of high amount of Lanthanum oxide in the samples which subsequently hinders the formation of single phase 1:13 material. These disadvantages can at least partially be attributed to the ball-milled powders used for LBM, which are not flowing well in the process due to the irregular shape and are highly oxygen sensitive due to the activated surface.

So, in this work, gas atomized powders were used, which have a spherical particle shape and are expected to be less sensitive to oxygen. The effect of temperature, time, cooling rate, and atmospheric condition of different heat treatment technologies on the microstructure of LBM processed LaFe_11.4_Si_1.2_Co_0.4_ was systematically investigated. The aim of this study was to determine if a high percentage of 1:13 phase and good magnetocaloric properties can be achieved after short-time annealing of LBM samples.

## 2. Materials and Methods 

**Figure 1:**
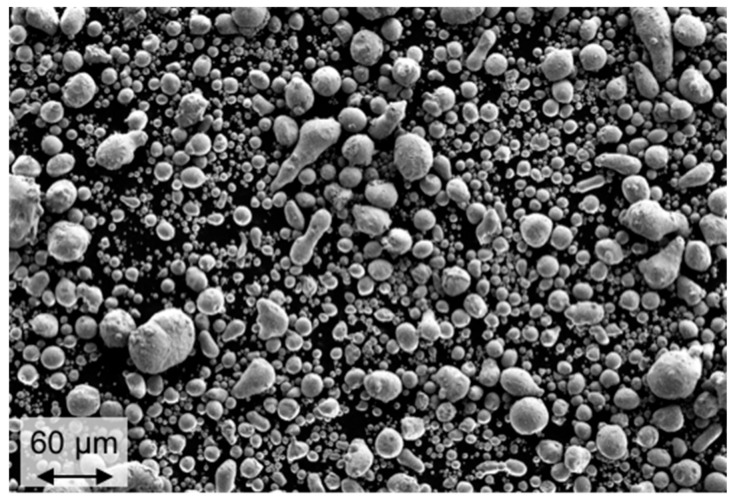
Powder morphology LaFe11.4Si1.2Co0.4 below 63 µm.

Powder for LBM-processing was synthesized by gas atomization under Argon inert gas atmosphere. The raw material of LaFe_11.4_Si_1.2_Co_0.4_ with a 15% La excess was molten and atomized at 1680 °C including superheating of 200 °C. A close-couple-atomizer nozzle was chosen to guarantee a narrow and fine particle size distribution within the desired range. Through careful selection of atomization parameters, the powder yield below 63 µm was increased from 40% to 75% depending on the gas-to-melt-ratio (GMR). To guarantee good powder flowability, tangential-gas was introduced into the near-nozzle zone to prevent particle recirculation and formation of satellite structures [20]. The obtained powders were sieved below 63 µm in order to be processable by means of LBM [21]. Figure 1 shows an example of the obtained particle morphology when sieved below 63 µm.

The LBM-processing was carried out in an EOS M 270 machine in Argon atmosphere (less than 0.1% oxygen). Suitable process parameters have been investigated varying the laser power, scan speed, scan strategy, hatch distance, and build platform temperature. The detailed description can be found in [21]. In this study, simple cylindrical shapes with an inner diameter of 2 mm, outer diameter of 5 mm and 10 mm in length were produced for the microstructure investigation. 

**Figure 2:**
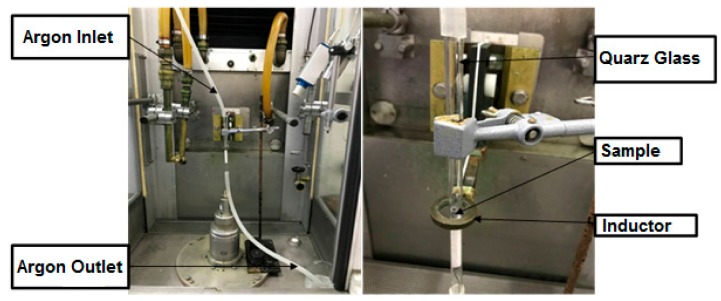
Set-up of induction heating system.

The samples were heat treated using two different heat treatment techniques: induction technology and conventional vacuum furnace. The induction experiments were carried out in an Induction hardening device of type VL 1000 of the company EFD Induktion GmbH. Due to the high oxygen sensitivity of Lanthanum, an oxygen-free system was set up and integrated into the induction system, which is shown in Figure 2.

The samples were placed in the center of the quartz tube on tempered glass fiber. Silicone tubes were attached to both sides of the tube. Before the heat treatment, argon (grade 5.0) was passed through the tube for about 1 hour to discharge the oxygen from the system and set a nearly oxygen-free atmosphere inside the tube.

**Figure 3:**
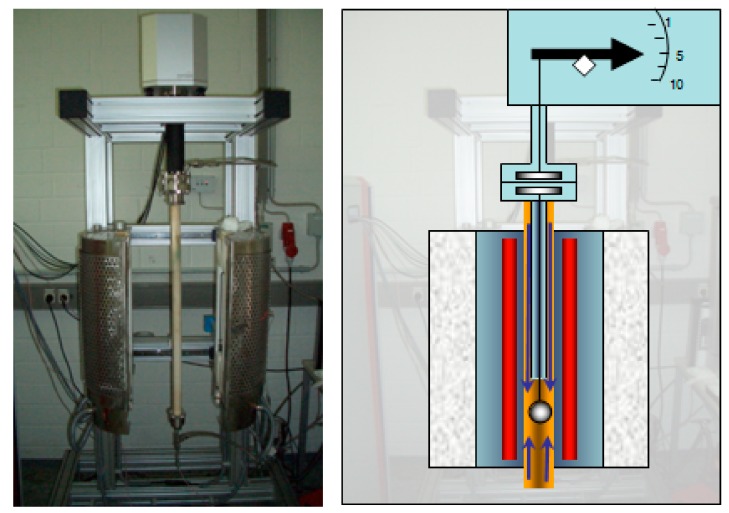
Thermogravimetric analyzer (Firm - Rubotherm). Left: picture with open furnace, Right: schematic structure.

The conventional heat treatment was carried out in thermogravimetric analyzer (TA-Instruments/Rubotherm TGA). An image of the schematic structure of the thermogravimetric analyzer is shown in Figure 3. The furnace wall is formed by an outer and an inner ceramic tube, the sample is suspended on a platinum wire. The connection of the vacuum pump is also located above the furnace. An external turbo vacuum pump was connected to the system to improve the vacuum in the system. After connecting the turbo pump, a vacuum in the range of ~ 2.0x10^-5^ mbar is achieved.

The samples were characterized regarding oxygen content (inert gas fusion method, Eltra Elemental Analysers ONH 2000) and microstructure. Metallographic sections were first prepared and examined for the structure in a scanning electron microscope (SEM) of the type JXA-8200 WD/ED combined microanalyzer. To ensure conductivity, these samples were steamed with carbon for 5 s after preparation. This ensured a layer thickness of less than 10 nm. The sample surfaces thus prepared were examined on an electron beam microprobe with a field emission cathode. High-contrast images were generated with an acceleration voltage of 10–20 kV and using the secondary electrons (SE) or back scattered electrons (BSE). Since the primary electrons are diffracted more at the atomic nucleus with increasing atomic number, the different phases could already be distinguished on the basis of their contrast with the backscattered electron detector. A quantitative determination of the chemical composition was done via Energy-dispersive X-ray spectroscopy (EDX). The magnetic properties were determined using a Microsense EZ9 vibrating sample magnetometer (VSM). By collecting isothermal magnetization curves around the Curie temperature, the magnetic entropy change ∆SM was calculated employing the Maxwell relation. Furthermore, the amount of α-Fe in the heat treated samples was estimated from thermomagnetization curves in 1 T field, comparing the magnetization above T_C_ to the magnetization of pure iron.

## 3. Results and Discussion

### 3.1. Metallographic Characterization by Microprobing

**Figure 4:**
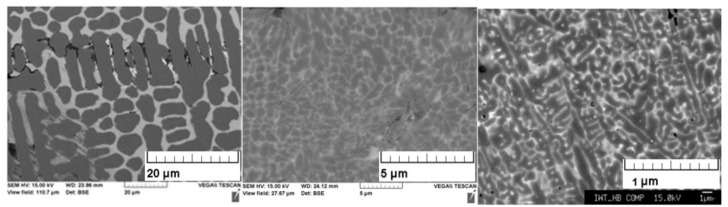
Microstructure of the ingot material (left), the gas atomized powder (middle), and the samples after the Laser Beam Melting (LBM) process (right)

The microstructure of the ingot material, the gas atomized powder, and the material after LBM are shown in Figure 4. As expected, the microstructure after rapid solidification during gas atomization and LBM is significantly refined compared to the cast ingot and is consisting of α-Fe and La-rich dendrites. The chemical composition, as well as the oxygen content, determined after the LBM process is given in Table 1. In comparison to the stoichiometric composition, the La content is slightly elevated and the Fe content is low. According to the results of the EDX analysis of the LBM sample, a certain gray microstructure around the black grains consisting of a small amount of 1:13 phase was found. The oxygen content of 0.5 wt. % is lower compared to the 0.9 wt. % reported in [19].

#### 3.1.1. Conventional Heat Treatment (in TGA Device)

**Figure 5:**
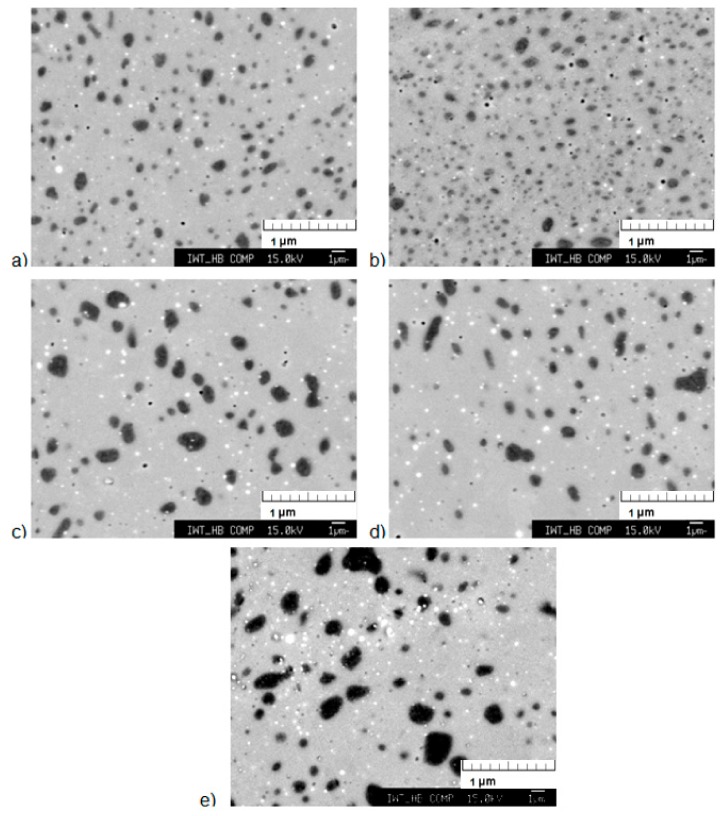
Scanning electron microscope (SEM) image of the samples heat-treated at 1373 K for: (**a**) 30, (**b**) 60, (**c**) 120, (**d**) 240, and (**e**) 480 min under vacuum in the thermogravimetric analyzer.

The LBM samples were heat treated in the conventional vacuum furnace under high vacuum condition. Figure 5 shows the results after heat treatment at 1373 K for 30, 60, 120, 240, and 480 min and quenching to room temperature. The evaluation of the metallographic results shows that already at a holding time of 30 min the dendrites formed in the LBM process have disappeared, a high percentage of 1:13 phase has formed and the α-Fe phase is evenly distributed throughout the sample. When increasing the heat treatment time, the α-Fe grains agglomerate, but the percentage of α-Fe does not seem to significantly decrease.

The percentage of α-Fe determined by magnetization measurement is given in Table 2. The results are in good agreement with the microstructure investigation since the values are in a similar range for all measured samples. According to these results, the percentage of α-Fe slightly decreases with the increase in heat treatment time from 30 min to 60 min. At higher holding times of 120 and 240 min, the values are higher compared to 60 min, but the variations are hardly significant. The lowest percentage of α-Fe that was reached, 25%, is high compared to the values reported in the literature for a successful heat treatment, where commonly values below 10% are achieved. Mayer et.al [12] reported 95 wt. % of 1:13 phase after annealing gas atomized LaCe(FeMnSi)_13_-alloy for 60 min at 1373 K. This can be attributed to oxidation during the heat treatment, due to which not enough lanthanum is available to form the 1:13 phase.

**Figure 6:**
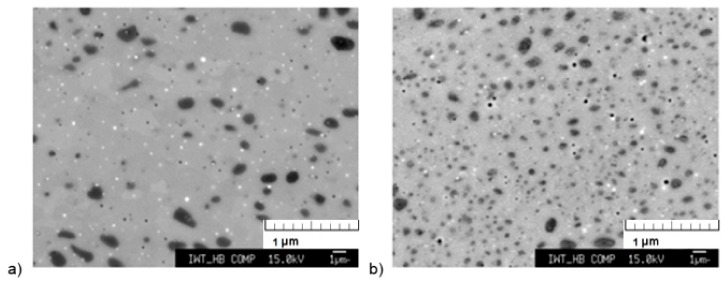
Comparison of the microstructure of the samples heat-treated at 1373 K, 60 min with different cooling rates: (**a**) 10 °C/min and (**b**) 65 °C/min under vacuum in the thermogravimetric analyzer.

Figure 6 shows the comparison of the microstructure of the samples which were cooled by rapidly cooling the ceramics tubes (65 °C/min) and furnace cooled (10 °C/min). A clear effect of the cooling method on the microstructure is visible. The sample which was furnace cooled to room temperature shows a greater amount of 1:13 phase compared to the sample that was rapidly cooled. According to the magnetization measurements, the percentage of α-Fe in the sample that was furnace cooled is around 20 wt. %, compared to 25 wt. % for the rapidly cooled sample as given in Table 2. The researchers in [22] investigated the influence of different cooling processes after high-temperature annealing of cast LaFe_11.6_Si_1.4_. Only a small influence of the cooling rate was reported and the highest amount of 1:13 phase was found after furnace cooling (95.3%), followed by air cooling (94%) and quenching in ice water (92.4%). 

#### 3.1.2. Induction Technology

**Figure 7:**
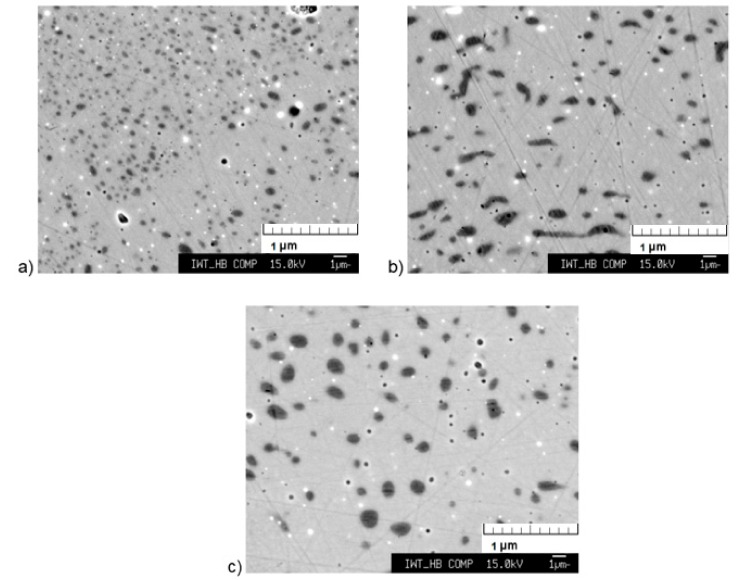
SEM image of the sample induction heat-treated at 1373 K for: (**a**) 15 min, (**b**) 30 min, and (**c**) 45 min.

The microstructure of the samples that were induction heat-treated at 1373 K for 15, 30, and 45 min soaking time in an argon atmosphere are shown in Figure 7. The microstructure after 30 min of heat treatment is similar to the microstructure of the sample which was heat treated conventionally at the same temperature and holding time, though the α-Fe grains are less spherical. By increasing the holding time of the induction heat treatment to 45 min, the form of the grains changes to ball-shaped, while the percentage of α-Fe is not significantly increased (Table 3). For a shorter heat treatment time of 15 min, the α-Fe grains are finer, and the highest percentage of 1:13 phase is achieved. Hence, compared to the conventional heat treatment, the optimum holding time is much shorter for the induction technology. It is also to be noted that the heating time required to reach the optimum temperature in induction technology is a few seconds only whereas conventional heat treatment takes several minutes (30 min) to achieve desired soaking temperature.

### 3.2. Magnetocaloric Properties

**Figure 8:**
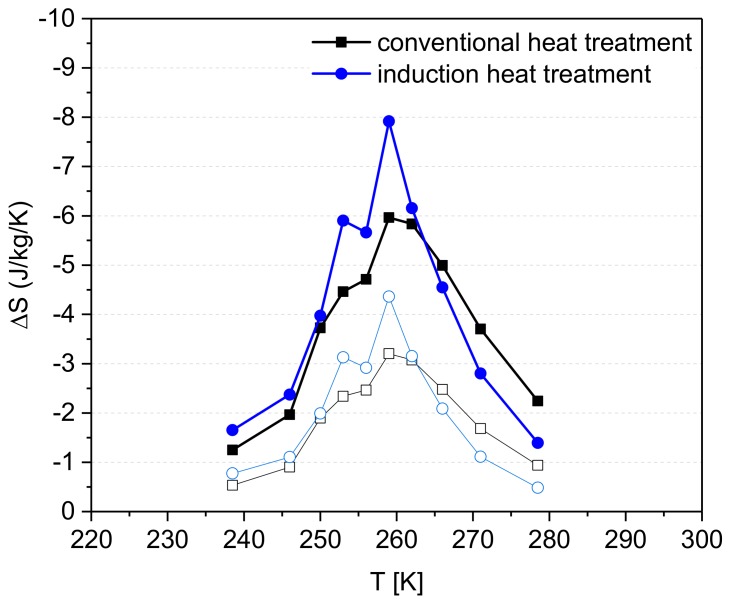
Magnetic entropy change (∆S) as a function of temperature for LaFe11.4Si1.2Co0.4 samples annealed by conventional heat treatment at 1373 K for 60 min (slow cooling) and by induction heat treatment at 1373 K for 15 min; magnetic field change is 0–1 T (open symbols) and 0–2 T (closed symbols).

The magnetic entropy change (∆S) was determined from isothermal magnetization data each for the sample with the highest amount of 1:13 phase heat treated with conventional and induction technology. The curves of ∆S versus temperature in a magnetic field change of 0–1 T and 0–2 T for the samples “conventional heat treatment–1373 K–60 min–slow cooling” and “induction heat treatment–1373 K–15 min” are shown in Figure 8. As expected due to the higher percentage of 1:13 phase, ∆S is higher for the induction heat treated sample. The maximum entropy change is -7.9 J/kg/K (0–2 T) at 259 K. For the conventional heat-treated sample, the maximum ∆S is -6.0 J/kg/K (0-2 T), at the same temperature. The values are compared to ∆S reported in [23] for LaFe_11.28_Si_1.2_Co_0.52_. With a volume fraction of 1:13 phase similar to this work (82%), a magnetic entropy change of about—6 J/kg/K was achieved for 2 T magnetic field change. However, the Curie-temperature determined in [23] is significantly lower (around 240 K), despite the slightly higher Co-content. In accordance with that, in [24] T_C_ of LaFe_11.4_Si_1.2_Co_0.4_ is around 245 K. The shift in the Curie-temperature could be attributed to the oxygen content and the percentage of the α-Fe present in the samples after the heat treatment [25]. It is well-known that T_C_ is proportional to x in La(Fe_1-x_Si_x_)_13_. So, if the overall content of α-Fe increases, the content of Fe in the 1:13 phase decreases, hence the Si-content and, respectively, T_C_ are increased. With an increased amount of oxygen, typically more La-oxide is formed, and La is not available for the formation of 1:13 phase. Hence, the content of α-Fe increases as well, and this leads to increase in T_C_.

## 4. Conclusions

Samples of the composition LaFe_11.4_Si_1.2_Co_0.4_ were produced by Laser Beam Melting from gas atomized powder. Due to the rapid cooling during the solidification process, the microstructure in the powder and after the LBM process was significantly refined compared to as-cast material. Employing a conventional heat treatment to the LBM samples, the maximum percentage of 1:13 phase was reached after 60 min of annealing time at a temperature of 1373 K, which is similar to the optimum heat treatment conditions reported by [12] for gas atomized powder. Slow cooling after the annealing step leads to a lower fraction of α-Fe compared to quenching. By employing an induction heat treatment to the LBM samples, the annealing time was even further reduced to only 15 min at 1373 K, resulting in around 83% 1:13 phase and a magnetic entropy change ∆S of −7.9 J/kg/K (0–2 T). Hence, after the LBM process, a magnetocaloric effect comparable to literature values is achieved after a significantly reduced annealing time.

A systematic variation of Co composition in LaFeSiCo-based alloy will be tried and tested for tuning the Curie temperature (T_c_) towards room temperature. Also, addition of Mn or Ce as the alloying element and adding a hydrogenation step will be part of future study to analyze the effect on the Curie temperature. Furthermore, different compounds of magnetocaloric materials will be considered to analyze the feasibility of the proposed manufacturing approach.

## Figures and Tables

**Table 1:** Chemical composition of LaFeSiCo based alloy used in this study.

	Chemical Composition				
	La (wt. %)	Fe (wt. %)	Si (wt. %)	Co (wt. %)	O (wt. %)
**Stoichiometric**	16.7	76.4	4.1	2.8	-
**LBM sample**	17.5	75	4.1	2.9	0.5

**Table 2:** Percentage of α-Fe in the samples heat treated in the thermogravimetric analyzer.

	1373 K			
	30 min	60 min	120 min	240 min
**α-Fe [wt. %]**	32	25	30	28

**Table 3:** Percentage of α-Fe in the induction heat treated samples.

	1373 K		
	15 min	30 min	45 min
**α-Fe [ wt. %]**	17	28	30
